# Wellness in medical education: definition and five domains for wellness among medical learners during the COVID-19 pandemic and beyond

**DOI:** 10.1080/10872981.2021.1917488

**Published:** 2021-05-04

**Authors:** Stephana J. Cherak, Brianna K. Rosgen, Alexa Geddes, Kira Makuk, Sanjana Sudershan, Caroline Peplinksi, Aliya Kassam

**Affiliations:** a Department of Community Health Sciences, Cumming School of Medicine, University of Calgary, Calgary, AB, Canada; b Department of Critical Care Medicine, Cumming School of Medicine, University of Calgary, Calgary, AB, Canada; c Office of Postgraduate Medical Education, Cumming School of Medicine, University of Calgary, Calgary, AB, Canada; d Office of Undergraduate Medical Education, Cumming School of Medicine, University of Calgary, Calgary, AB, Canada

**Keywords:** Medical learner, wellness, well-being, medical education, COVID-19

## Abstract

**Problem**: The novel coronavirus SARS-CoV-2 disease (COVID-19) impacted medical learner well-being and serves as a unique opportunity to understand medical learner wellness. The authors designed a formal needs assessment to assess medical learners’ perspectives regarding distress related to disrupted training environments. This Rapid Communication describes findings from a qualitative study which defined medical learner wellness and validated five wellness domains.

**Approach**: We conducted follow-up telephone interviews to an online needs assessment survey to identify a learner definition for wellness and to validate five wellness domains, including social, mental, physical, intellectual, and occupational wellness. Using purposive and maximal variation sampling, 27 students were interviewed from July–August 2020. Thematic analysis was performed using a deductive thematic approach to qualitative analysis.

**Outcomes**: Medical learners defined wellness as a general [holistic] sense of personal well-being – the opportunity to be and to do what they most need and value. Learners validated all five wellness domains for medical education. Learners acknowledged the need for an adoptable and adaptable holistic framework for wellness in medical education.

**Next steps**: We recommend academic medical institutions consider learner wellness a key component of medical education to cultivate learners as a competent collective of self-reliant, scholarly experts. We encourage evaluation of wellness domains in diverse medical learner populations to identify feasible interventions potentially associated with improvements in medical learner wellness.

## Problem

Medical schools are called to help their progressively diverse student cohorts enhance their self-actualization and professional impact both during training and as future healthcare professionals [1]. We, among others [2], are concerned about medical learner [psychological, social and physical] well-being. In recent years, a number of medical schools have redesigned medical education including intellectual (e.g., learning, teaching and assessment) and occupational (e.g., professional development) wellness as core competencies of learning [3]. Though these efforts teach learners about wellness in current and future practices as physicians and healthcare professionals, they do not acknowledge a holistic framework for learner wellness [4], and do not address synergies among wellness domains associated with improvement in medical learner well-being [5]. Holistic wellness strategies and supports for medical learners are likely to merge many wellness domains.

The novel coronavirus SARS-CoV-2 disease (COVID-19) impacted medical learner well-being [6,7]. While the majority of medical learners acknowledge the enormous goodwill and timely efforts of their academic medical system, as individuals they felt inadequately prepared to continue their program to meet national standards and training requirements [8]. Our contention is that without targeted wellness interventions for specific medical learner programs and systems to address individual wellness topics for learners, wellness consequences among medical learners will perpetuate through the next generation of healthcare professionals. Such wellness interventions, however, require a set of core wellness domains to support wellness intervention development, implementation and evaluation in a way that is feasible and valuable for learners [9].

Herein we discuss findings from semi-structured telephone interviews during COVID-19 that engaged learners to define medical learner wellness and to validate five domains of wellness [10].

## Outcomes

Using purposive and maximal variation sampling, 27 medical learners (who indicated interest on an online needs assessment survey) were interviewed via telephone from July–August 2020. Learners identified primarily as women (*n* = 18, 67%) and were mostly MSc or PhD graduate students (*n* = 14, 52%), followed by undergraduate medical students (*n* = 5, 19%), undergraduate bachelor’s students (*n* = 5, 19%), and postgraduate resident physicians (*n* = 3, 11%). Learners were asked how they define wellness and to validate five domains of wellness considered important by the study team. Thematic analysis was performed using a deductive thematic approach to qualitative inquiry. Additional descriptions of methods and outcomes are provided in the Supplemental Materials.

Learners defined wellness in medical education commenting on a general sense of personal well-being, the opportunity to be what is most valued, and the opportunity to do what is most valued. Learners validated a set of five wellness domains that included social, mental, physical, intellectual, and occupational wellness. In Table 1 we include example quotations.

**Table 1.** Definitions and example quotations, from semi-structured interviews exploring medical learners’ perceptions and experience of wellness in medical education during the COVID-19 pandemic, University of Calgary, Alberta, July–August 2020.^a^

Learner definition for learner wellness in medical education: A general sense of personal well-being – the opportunity to be and to do what is perceived by the learner as most needed and most valued.
*General sense of personal well-being* ‘I would describe wellness as a general and personal sense of well-being. I think being “well” is a concept is that always changing and evolving, at times good and other times bad, and really something that should be considered on a personal level and supplemented by other levels. So, as a learner, how I might describe wellness now is totally different than how I might describe wellness tomorrow, or even from yesterday, but … generally it’s a sense of personal and holistic well-being.’ (117–84)
*The opportunity to be what is most needed and valued* ‘A learner who recognizes that wellness is important, perhaps most, takes the time to figure out how to make wellness happen for them in whatever way that means. Asking for flexibility and asking for help so that they can truly be well – reaching out is a big thing. I also think that [a well] learner has a good learning support system, from peers to [supervisors] to [program] coordinators and administrators. Being a learner, we have two homes – our personal home and our academic home. So, if we think about a learner who is well, we can think about being well … in both of our homes.’ (117–13)
*The opportunity to do what is most needed and valued* ‘Perhaps … I think that … there are two different “facets” of wellness that both fit in to the overall definition of what it means to be well. […] As a medical learner it’s easy to do more – publish more, volunteer extra hours, spend more times on projects you know aren’t going to be fruitful to you. During clerkship, there were times when I didn’t eat for 26 hours, didn’t go to the bathroom. What I’m getting at is that yes, it’s easy to do more, especially in medical education. What’s the difference-maker is whether doing more is determined by the learner as important. Do I feel needed? Are my contributions valuable? Allowing the learner to answer those questions is fundamental in ensuring good wellness for all.’ (117–24)
Five wellness domains for wellness interventions in medical education
*Social wellness: Domain of wellness in which medical learners are a part of, and are accepted by their social environment in medical education, and are comfortable expressing their feelings, needs, identities and opinions to colleagues and instructors* ‘Social wellness is different to everyone – but it’s the one thing we all have in common because everyone needs social connectivity. Med[ical] students might need it more – demands of medical education can be isolating. Social wellness in medical education … a sense of belonging … having one-to-one connections that are consistent and authentic.’ (117–69)
*Mental wellness: Domain of wellness in which medical learners realize their own potential, cope with the normal stressors of life, work productively and fruitfully, and make meaningful contributions to their personal and professional communities* ‘I think of mental wellness as a genuine commitment to my communities. Being mentally well means showing up as a contributing, compassionate and empathic member of my personal and professional communities in a way that is meaningful to my mental well-being and stimulating for others. […] I think that if medical students were given the support and flexibility and the time to be committed to our communities in these ways, we could be a great deal more. I know that I would be able to better realize my full potential about if I had this extra space to be mentally well.’ (117–76)
*Physical wellness: Expectation of wellness of a leaners’ body, including the active and continuous effort to maintain optimum levels of physical activity and focus on nutrition, as well as self-care and maintenance of a healthy lifestyle* ‘Physical wellness is keeping my body physically fit and nourished so that I am able to be the best person that I can be. So, although this sounds like a “doing” thing, it’s more of a “being” thing, for learners … in my opinion. I don’t view exercise as a way to burn calories but rather a means to move my body so that every other domain of wellness can be fulfilled and impacted positively. It’s a way to fill up my cup, so in this sense, it’s really about maintaining good overall health.’ (117–83)
*Intellectual wellness: Domain of wellness in which medical learners are enabled to pursue creative, mentally-stimulating activities that expand their knowledge, develop skills, and foster life-long learning and teaching towards self-actualization* ‘Intellectual wellness is everything to do with learning – my education. Other activities such as reading a book or taking part in an unrelated seminars would fit here, even if those things are a “break” from my thesis, I think they still contribute to me being intellectually well. Also, having the time to give my brain a break. To make sure I continuously love learning.’ (117–05)
*Occupational wellness: Protection and promotion of medical learners, by preventing and controlling occupational diseases and accidents, by elimination of conditions hazardous to health and safety, and by the development and promotion of healthy and safe working environments and organizations* ‘Occupational wellness to me is regarding working – doing. I want to know that I am safe, generally, that I am given the conditions and support to work effectively, and there aren’t barriers in the workplace that prevent me from taking care of myself or having time to myself in order to be well. The culture [of healthcare] is not one of self-care, ironically.’ (117–54)

^a^‘[…]’ indicates that text has been omitted, while ‘ … ’ indicates a pause in speech.

### Learner definition for learner wellness in medical education

Here we provide the learner definition for learner wellness in medical education, as ‘a general sense of personal well-being – the opportunity to be and to do what is perceived by the learner as most needed and most valued.’

#### A general sense of personal well-being

Learners highlighted the value of a general (i.e., holistic) sense of personal well-being while enrolled in medical education. They reported that they valued peer support (both giving and receiving), actively employed personal coping strategies for positive adaptations to stress and engaged in informal physical health promotion initiatives among their classmates. They also frequently engaged in opportunities to cultivate a holistic sense of intellect (i.e., intellectually stimulating activities aside from their program of learning) and to develop professionally (e.g., learner-physician mentorships). Nonetheless, nearly all students noted that the current culture of medical education does not ensure safe and health-promoting cultures of learning and training in medical education. Seen from the eyes of learners, medical education does not provide adequate training on personal well-being and how to effectively manage their personal well-being concerns. Although most participants felt usually overwhelmed (e.g., they [especially more junior learners] were challenged with finding a sense of balance) and experienced frustration generally (e.g., they struggled with adjusting to new and changing schedules), they stated they understood the structure of their education program was appropriate for enabling medical learners to succeed in their training as future professionals in healthcare. Learners who participated in our study were especially concerned about opportunities to be and to do what they felt, as learners, is most needed and valued – a component of their definition of wellness.

#### The opportunity to be what is most needed and valued

Learners acknowledged a disconnect between ‘being’ (i.e., socially, mentally, physically) and ‘doing’ (i.e., intellectually, occupationally) what is perceived to be important – needed and valued – in medical education. Learners suggested that part of the solution was to ensure two-way communication between these disciplines, in order to acknowledge the synergies among the domains that comprise them. Many participants described the often rapidly effaced boundaries between domains of wellness in medical education and articulated that promoting better dialogue on different opportunities ‘to be’ and ‘to do’ would ensure the development of realistic, usable, friendly, and effective wellness interventions. Learners provided specific recommendations, which included having small group activities unrelated to medical education (i.e., without defined criterium or objectives to study) and dedicated specifically to peer-to-peer interaction (e.g., support, comradery); expanding on current opportunities for mental wellness (e.g., ensuring that hours to access mental wellness services align with the often-inflexible hours of training in medical education); and developing formal physical wellness programs and initiatives (e.g., run clubs, workout buddy systems).

#### The opportunity to do what is most needed and valued

Learners desired more opportunities in medical education to do (i.e., intellectually and occupationally) what is most needed and valued to them. Prominent suggestions included finding ways to offer more personalized assistance with learning (e.g., one-on-one teaching and training sessions specific to learner program and stage of training) and having tailored or personalized professional development workshops to meet desires of medical learners at all levels of medical education (i.e., younger students desired higher-level [superficial] professional development while more senior students desired detailed and action-oriented plans). Even participants who felt satisfied in their opportunities ‘to do’ recognized attaining this level of satisfaction took personal strategies developed on an individual level, rather than promoted from their program and academic medical system. Participants generally attested that opportunities for learners to attain intellectual and occupational wellness in medical education are well-organized and available.

### Five domains for wellness in medical education

We responded to a call to action at our academic medical institution for enhanced learner wellness by asking learners themselves to take part in validating wellness domains important for medical learners. Participants validated social, mental, physical, intellectual, and occupational wellness as important for consideration in future wellness initiatives. Learners commented that these domains are important based on the process, format, and outcomes of medical education. In Table 1 we provide definitions and example quotations for learner-validated wellness domains.

## Next steps

Our findings present a framework for structuring wellness initiatives in medical education in response to the COVID-19 pandemic and beyond. We report a learner-defined definition for wellness and five domains of wellness that learners validated to be important. The data is based on a needs assessment of medical learner programs at a single academic medical institution in Alberta, Canada. In our experience, the lessons learned from the COVID-19 pandemic can serve as a foundation on which wellness interventions can build. Engaging and empowering medical learners in structuring their own wellness to address underlying sources of individual medical learner distress than reactively reducing manifestations of stressors will allow learners to be wellness ambassadors with skills and confidence to encourage their medical colleagues to promote individual wellness. It is anticipated that endorsing proactive wellness interventions for learner wellness is applicable to future unprecedented events disruptive to medical education.

Given well-being is universal to learners enrolled in all medical schools, we recommend that medical educators in any academic system apply our results to medical learners generally. We have validated five domains that medical learners consider important that can be integrated within future multi-component wellness initiatives to address medical learner well-being. These domains can help to identify unmet wellness needs among individual learners within medical education programs. We encourage broadened evaluation of our findings to explore barriers to wellness domains that might include social isolation, general uncertainty, and distress, among others. Lack of preparedness regarding the shift to online learning is especially important during COVID-19. Above all, we hope that others will join in the quest to improve learner wellness in medical education – surely unchallenged with a gallant sprint.

## Supplementary Material

Supplemental Material
