# Supplementary material for: Wellness in medical education: definition and five domains for wellness among medical learners during the COVID-19 pandemic and beyond
Source: Med Educ Online. 2021 May 4;26(1):1917488. doi: 10.1080/10872981.2021.1917488 (PMC8097384; doi:10.1080/10872981.2021.1917488)
Supplement: Supplemental Material [file ZMEO_A_1917488_SM8790.docx]

**Supplemental Material**

**Approach**

**Wellness Scoping Review**

We conducted a comprehensive scoping review (*In Press, Cherak et al., [2021] CMAJ Open*) to synthesize diverse literature on interventions for enhanced medical learner wellness to informed medical educators of key components for rigorous, evidence-based wellness interventions to improve medical learner well-being. The observed heterogeneity from our review indicated that Canadian academic medical systems are not consistently implementing wellness interventions for enhanced medical education learning environments. Though we reviewed in detail hundreds of articles, we identified that only a small subset evaluated wellness interventions empirically using valid and reliable assessment tools. From our findings we recommended that medical education research on learner wellness would benefit from studies with rigorous design, reproducible methodologies and objective data collection. Through lines of inquiry for redesign of holistic medical learner well-being we suggested that future studies include graduate science education students (i.e., MSc, PhD students conducting scientific medical research), assess various domains of wellness in a sequentially adaptive manner to determine synergies, and evaluate trajectories of medical learners to know which wellness domains are impacted at different stages of medical teaching and training.

**Needs Assessment Phase I**

In phase I,^1^ 540 students at a large Canadian medical school participated in an online survey (April-June 2020) based on the needs assessment framework for learner assessment in medicine developed from the International Association for Medical Education.^2^ We used items with respect to Wellness Innovation Scholarship for Health Professions Education and Health Sciences (WISHES) framework domains^3^ and factors impactful to learners’ training from our scoping review of interventions for improved wellness of Canadian medical learner (under peer-review). The survey consisted of 21 discrete answer questions (including Likert-scale items) and open-ended responses within three sections: 1) demographics; 2) factors impactful for learners to thrive or struggle in their training during the COVID-19 pandemic; and 3) assessment of learner wellness domains regarding the COVID-19 pandemic. The objective of phase I was to understand the impact of the COVID-19 pandemic on learners in health sciences and medical education.

**Needs Assessment Phase II, Semi-Structured Interviews**

Learners involved in phase II consisted of those who indicated interest in completing a follow-up interview after the first phase of our needs assessment. In phase II, we completed formal, in-depth telephone interviews with learners to determine a definition for wellness along with a validated set wellness domains. We applied purposive and maximal variation sampling (i.e., sampling based on learner program and male/female) to recruit 27 students to participate in phase II interviews. Students did not receive compensation or reimbursement for taking part in the interview. On obtaining written consent, a research assistant with qualitative research experience (S.C.) conducted one-on-one telephone interviews over a 2-month period (July-August 2020) using a semi-structured interview guide (Supplemental Table 1). The interview guide was revised for brevity and clarity based on feedback from pilot testing with learners enrolled in each learner program in the Cumming School of Medicine. Interviews were reviewed on an ongoing basis and conducted until thematic saturation was achieved (i.e., until we well understood learners’ insights and no new ideas were presented). Interviews ranged from 22 to 42 minutes (mean 30.2 minutes, median 30 minutes) and were audio recorded and transcribed verbatim. Immediately following each interview S.C. also took field notes.

Thematic analysis of the interviews was performed in duplicate through iterative constant comparison to allow an inductive, open approach to data acquisition for deeper understanding to emerge through careful consideration of the data. We applied engaged scholarship to thematic analyses; two learners graduated from or currently representing each of the learner programs (S.C., K.M., S.S., A.G.) analyzed and coded full data independently using NVivo Qualitative Data Analysis Software Version 12 (QSR International). Dyads had an average interrater agreement of 93% and discrepancies were resolved by in-depth discussion. Our unique engaged scholarship approach to data analysis and interpretation yielded a substantive understanding of learners’ definitions, perceptions and experience of learner wellness in medical education.

**From learners, to learners**

We received an outstanding amount of learner-to-learner advice and support for finding balance in medical education during times of struggle and disrupted training. Our findings suggest that medical learners are motivated to promote dialogue on wellness and well-being. We share below four key questions and points of consideration from learners, to learners.

**“What first interested you about medical education?”**

Use specific language and describe what is interesting or exciting to you. Home in on the excitement that brought you to medical education in the first place—keeping hold of the why will help you to do the what.

**“What is your personal motivation for learning medicine?”**

Talk sincerely to yourself about how medical education will help you develop certain personal skills, and why you think a profession in healthcare fits your personal style. Consider why you think the culture of medical education matches your own personal values.

**“How does medical education align with your career goals?”**

Ambition and motivation in medical education helps to consolidate your enthusiasm and willingness to work hard. Think about classmates or instructors who have inspired or directed you towards your desired career path of a healthcare professional, and how that path aligns with your own personal work.

**“Be practical, and grounded.”**

Whilst it is important to be excited and motivated about being a medical learner, focus on small, achievable results for a defined period of time (e.g., a certain course or clinical clerkship). Consider setting SMART [specific; measurable; achievable; realistic; timely] goals to recognize results in personal and professional development.

**Supplemental References**

**1.** Cherak S, Brown, A., Kachra, R., Makuk, K., Sudershan, S., Paget, M., & Kassam, A. Exploring the impact of the COVID-19 pandemic on medical learner wellness: a needs assessment for the development of learner wellness interventions. Canadian Medical Education Journal. . 2021.

**2.** Pangaro L, ten Cate O. Frameworks for learner assessment in medicine: AMEE Guide No. 78. Med Teach. 2013;35(6):e1197-1210.

**3.** Kassam A, Ellaway R. Acknowledging a Holistic Framework for Learner Wellness: The Human Capabilities Approach. Acad Med. 2020;95(1):9-10.
